# Nuclear localization of Pretaporter in *Drosophila melanogaster* third-instar larvae salivary gland and its deficiency-associated nuclear phenotype

**DOI:** 10.17912/micropub.biology.001246

**Published:** 2024-08-13

**Authors:** María Constanza Silvera, María José Ferreiro

**Affiliations:** 1 Current affiliation: Departamento de Neurofisiología Celular y Molecular, Instituto de Investigaciones Biológicas Clemente Estable, Montevideo, Uruguay; 2 Past affiliation: Departamento de Biología del Neurodesarrollo, Instituto de Investigaciones Biológicas Clemente Estable, Montevideo, Uruguay; 3 Current affiliation: Departamento de Neurofarmacología Experimental, Instituto de Investigaciones Biológicas Clemente Estable, Montevideo, Uruguay

## Abstract

The *Drosophila melanogaster* protein Pretaporter, is thought to reside in the endoplasmic reticulum and relocate to the plasma membrane during apoptosis. However, very little is known about its subcellular distribution in different cell types and conditions. Here, we present the first report of Pretaporter´s subcellular distribution in the salivary gland cells of *Drosophila* third-instar larvae, finding it enriched in cell membranes, apical granules, and unexpectedly within cell nuclei. Pretaporter loss-of-function mutants exhibited hypotrophied nuclei, suggesting a potential role in DNA endoreplication control. These findings prompt a reevaluation of Pretaporter’s functions and encourage future research aimed at unraveling novel roles.

**Figure 1. Pretaporter expression pattern in Drosophila wild-type L3 salivary gland and nuclear phenotype in prtp null mutants.**
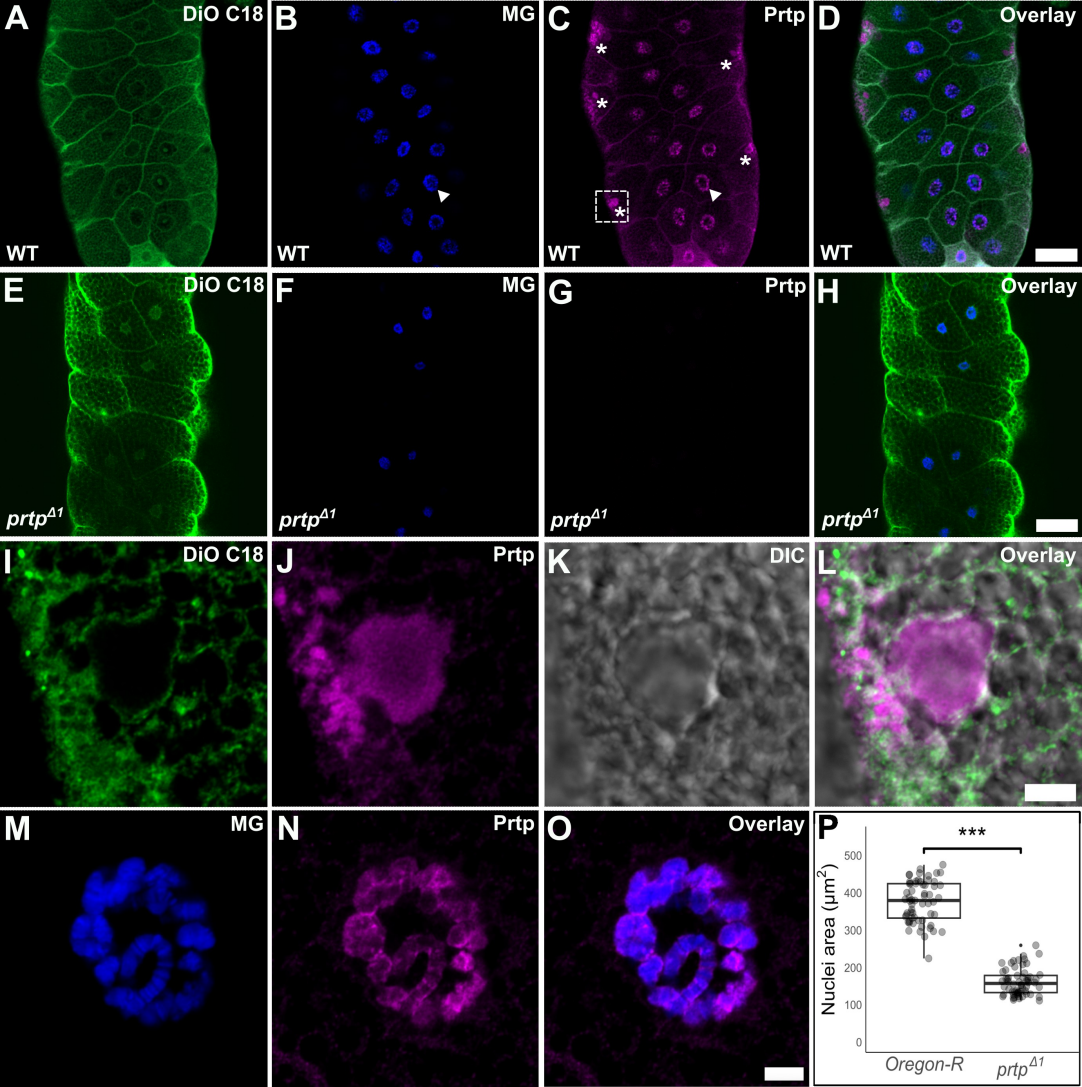
**A-D.** Representative image showing a single confocal section of a wild-type salivary gland stained with DiO C18 (green) to label membranes, methyl green (blue) to label nuclei, and anti-Prtp (magenta). The Prtp-immunopositive staining was observed in cell membranes, nuclei and clusters of granule-like profiles (asterisks). 20x objective. Scale bar: 50 µm. **E-H.** Single confocal section of *prtp* loss-of-function mutant ( *prtp ^Δ1^* ) salivary gland (negative control). No Prtp-positive fluorescence was observed. 20x objective. Scale bar: 50 µm. **I-L.** Magnification of white dotted box in C showing a large and smaller Prtp positive apical SG-like structures. DIC: Differential interference contrast. 63x objective and 3x digital zoom. Scale bar: 5 µm. **M-O.** Magnification of the nucleus indicated with white arrowheads in B and C. 63x objective and 3x digital zoom. The immunofluorescent signal appears to represent a very thin filamentous structure intimately associated with the large polytene chromosomes typical of the salivary gland at this larval stage. This potential association between Prtp and DNA from polytene chromosomes should be explored in depth by further experiments. Scale bar: 5 µm. **P.** Quantification of wild-type Oregon-R and *prtp ^Δ1^* nuclear size of salivary gland cells. Mann–Whitney–Wilcoxon test p < 0.001 (***). N = 10 nuclei per individual, 6 individuals.

## Description

The *Drosophila melanogaster* gene *pretaporter* ( *prtp* ), homologous to the human protein Erp46, encodes a disulfide-isomerase protein, Pretaporter (Prtp), that has three thioredoxin-like central domains, an N-terminal signal peptide and a C-terminal signal for endoplasmic reticulum (ER) retention. It has been suggested that Prtp is located in the lumen of the ER and translocates to the plasma membrane under apoptotic stimuli that normally occur through development, where it acts as a ligand for the phagocytic receptor Draper, the human MEGF10 homologue (Kuraishi et al., 2009; Nakano et al., 2019) . Additionally, Prtp could have a role in protein folding, as suggested by bioinformatics inferences (Stable PanTree Identifier: PTN005231188). High throughput studies indicated that *prtp* is expressed in most *Drosophila* tissues across its life cycle (Chintapalli et al., 2007; 2013; Robinson et al., 2013) . In particular, *prtp* is expressed in the salivary glands of third-instar larvae (L3), an organ frequently used to study secretion (Loganathan et al., 2021) .

Late in larval life, the salivary gland cells of L3 larvae contain a particular type of secretory granules (SG) which originate from the Golgi apparatus, accumulate in the apical region, gradually grow by fusion of smaller granules of less than 0.3 µm making larger granules of up to 7 µm in diameter which finally are secreted into the gland’s lumen (Rizki, 1967; Farkas and Sutakova, 1998; 1999) . Some of the proteins that act in endolysosomal pathways required for the biogenesis and maturation of SG include the vesicle coat protein Clathrin, the Clathrin adaptor protein complex 1, and Syntaxin 16, a member of the SNARE family (Chen et al., 2010; Burgess et al., 2011; 2012; Ma et al., 2020) . Interestingly, data from the Reactome pathway database suggest that Prtp could be involved in vesicle-mediated transport, trans-Golgi network vesicle budding and lysosome vesicle biogenesis, including Prtp in the Lysosome Cargo protein data set together with Clathrin, Clathrin adaptor protein complex 1, and a SNARE family member, the Vesicle-associated membrane protein 7 (Stable Identifier: R-DME-435030) (Reactome Pathway Database, 2024) . Moreover, knocking down another member of the SNARE family in *Drosophila* intestinal stem cells, the ADP Ribosylation Factor 1, resulted in upregulation of Prtp, with the former postulated to function downstream of the first (Aggarwal et al., 2022) .

Very little is known about the subcellular distribution of Prtp in specific cell types or tissues and at different physiological conditions and developmental stages. Here we use immunohistochemistry and laser confocal microscopy to investigate the localization of Prtp in the cells of *Drosophila melanogaster* larvae`s salivary gland at a developmental stage when they have intense secretory activity (Rizki, 1967; Farkas and Sutakova, 1998) .

In wild-type (WT) larvae, Prtp was observed in membranes and in clusters of apically located granular profiles with the same subcellular localization and size of larger secretory granules (diameter: 6.3 ± 2.9 µm, n=15 granules of 6 individuals) ( **Figure 1C -D, and I-L** ). Surprisingly, we also found enrichment of Prtp-positive fluorescence in the cell nuclei ( **Figure 1C** ). These nuclei are normally very large as a result of repeated rounds of DNA replication resulting in extremely large polytene chromosomes (Smith and Orr-Weaver, 1991) , which are readily visible under the microscope (For a recent review see Zhimulev et al., 2024). No Prtp immunohistochemical reaction was observed in the salivary gland of larvae carrying a Prtp loss-of-function mutation, *prtp ^Δ1^* ( **Figure 1G** ). High resolution visualization of Prtp in WT nuclei suggested that this protein forms a scaffold-like pattern surrounding chromosomes ( **Figure 1M -O** ). This prompted us to examine the cell nuclei of salivary glands in *prtp ^Δ1^* mutants ( **Figure 1F,H and P** ). In these mutants the nuclei size was markedly reduced with respect to that from WT ( **Figure 1P** , p<0,001).

Our observation of Prtp localization in structures that probably correspond to large secretory granules at a developmental phase of rich glandular secretion constitutes the first experimental evidence in favor of Prtp potentially playing a role in the processes of vesicular transport, Golgi budding, and endolysosomal trafficking. Prtp involvement in this process should be investigated by further experiments, employing specific markers and a time-lapse evaluation.

Nuclear localization of Prtp in *Drosophila* cells has so far not been reported but there is evidence for nuclear localization of the human homologue Erp46 in a renal cell carcinoma cell line (Duivenvoorden et al., 2014) . This localization suggests that upon certain conditions, an intracellular cleavage of the C-terminal signal for ER retention allows for the nuclear enrichment of Prtp. This possibility should be addresed by further experiments. Cell nuclei in the *Drosophila* salivary gland grow rapidly by means of several rounds of endoreplication (Smith and Orr-Weaver, 1991; Ovrebo and Edgar, 2018) and thus the hypotrophied nuclear phenotype reported here suggests that Prp might contribute to the control of DNA endoreplication. Another possibility is that nuclear DNA reduction in *prtp* mutants could be due to a developmental delay with respect to WT larvae, a possibility which could be investigated with a timed re-evaluation of this parameter in future experiments. Additional analyses are necessary to fully understand the nuclear function of Prtp in *Drosophila* salivary gland.

These findings prompt a reevaluation of Prtp's cellular functions and open avenues for future research aimed at unraveling its multifaceted roles.

## Methods

***Drosophila melanogaster stocks***

We used the following *Drosophila melanogaster* strains: wild-type *Oregon-R* and *w ^1118^ prtp ^Δ1^* , carrying a loss-of-function allele of *prtp* as negative control (kindly provided by Dr. Nakanishi, Kanazawa University, Japan) (Kuraishi et al., 2009) . Flies were raised in standard culture medium at 25°C, and a 12-12h light-darkness regime.

***Immunohistochemistry***

Salivary glands of wandering L3 male larvae from each strain (n= 6 individuals per genotype) were dissected in cold PBS 1X, fixed at room temperature in 4% paraformaldehyde in PBS 1X for 30 minutes, washed 3 times in PBS 1X, permeabilized in PBS-TritonX-100 0.5% (PBS-T) during 15 minutes and incubated in a 1/300 dilution of rat anti-Prtp primary antiserum (kindly provided by Dr. Nakanishi, Kanazawa University, Japan; Kuraishi et al., 2009) for two hours at 4°C. Samples were then washed 3 times in 0.5% PBS-T and incubated with goat anti-rat-Cy3 secondary antibodies (Molecular Probes, 1:1000 dilution), cell membranes were labelled with the lipophilic marker DiOC18 (Invitrogen, 1:1000 dilution) and cell nuclei were labelled with a 1:5000 dilution of DNA marker methyl green (Prieto et al., 2014) . Finally, the samples were washed 3 times in PBS-T0.1%, twice with PBS 1X and mounted in Tris-Glycerol 80% pH=8.8.

***Confocal microscopy, image analysis and statistics***

Laser confocal microscopy was done with a ZEISS 800 AiryScan LSM (IIBCE platform), using a 488nm laser to visualize DiOC18, a 561nm laser to visualize the Cy3 fluorophore and a 640nm laser to visualize methyl green. Panoramic images of salivary gland were captured using a 20x objective (NA=0.5) and high-resolution images were taken using an oil-immersion 63x objective (NA=1.4) and 3x digital zoom. Images were analyzed using the open-access software FIJI/ImageJ (Schindelin et al., 2012) . Nuclei area was quantified for individual nuclei by measuring MG fluorescence area (N = 10 nuclei per individual, 6 individuals). The statistical analysis and graphical plot were generated using RStudio open access software ( http://www.rstudio.com ). Shapiro-Wilk test was used to evaluate normality of nuclei area data (Shapiro, Wilk and Chen, 1968) . Due to the non-compliance with this fundamental precept for the use of a parametric statistical test, we carried out the statistical analysis of the comparison between groups using the non-parametric Mann-Whitney-Wilcoxon test (Mann and Whitney, 1947) .

## Reagents

**Table:** 

**ANIMALS**	**GENOTYPE**	**AVAILABLE FROM**
*Drosophila melanogaster*	X*/X* or Y; +/+; +/+ (Oregon R)	Dr. Goñi, UdelaR, Uruguay
*Drosophila melanogaster*	* X ^w1118prtpΔ1^ /X ^w1118prtpΔ1^ or Y; +/+; +/+ * (prtpΔ1)	Dr. Nakanishi, Kanazawa University, Japan

**Table:** 

**ANTIBODY**	**ANIMAL AND CLONALITY**	**DESCRIPTION**
anti-Prtp	Rat	Anti-Prtp antibody was raised by immunizing rats with GST-fused Prtp that had been expressed in *E. coli* and puriﬁed to homogeneity. (Kuraishi et al., 2009)
Secondary antibodies	goat anti-rat-Cy3	Molecular Probes inc.

**Table:** 

**Other reagents**		**DESCRIPTION**
PBS 1X	Phosphate-Buffered Saline	Merck. To make 1 L of PBS, add 100 mL of 10X PBS to 900 mL of water. This PBS recipe contains 137 mM NaCl, 2.7 mM KCl, 10 mM Na2HPO4, and 1.8 mM KH2PO4.
PBS-T	PBS-TritonX-100 0.5% and 0.1%	PBS 1x with detergent
DiOC18	lipophilic marker	Invitrogen #D275
Methyl green	DNA marker	Prieto et al., 2014
Tris-Glycerol	80% pH=8.8.	Sigma-Aldrich #818709

## References

[R1] Aggarwal P, Liu Z, Cheng GQ, Singh SR, Shi C, Chen Y, Sun LV, Hou SX (2022). Disruption of the lipolysis pathway results in stem cell death through a sterile immunity-like pathway in adult Drosophila.. Cell Rep.

[R2] Burgess J, Jauregui M, Tan J, Rollins J, Lallet S, Leventis PA, Boulianne GL, Chang HC, Le Borgne R, Krämer H, Brill JA (2011). AP-1 and clathrin are essential for secretory granule biogenesis in Drosophila.. Mol Biol Cell.

[R3] Burgess J, Del Bel LM, Ma CI, Barylko B, Polevoy G, Rollins J, Albanesi JP, Krämer H, Brill JA (2012). Type II phosphatidylinositol 4-kinase regulates trafficking of secretory granule proteins in Drosophila.. Development.

[R4] Chen Y, Gan BQ, Tang BL (2010). Syntaxin 16: unraveling cellular physiology through a ubiquitous SNARE molecule.. J Cell Physiol.

[R5] Chintapalli VR, Wang J, Dow JA (2007). Using FlyAtlas to identify better Drosophila melanogaster models of human disease.. Nat Genet.

[R6] Chintapalli VR, Wang J, Herzyk P, Davies SA, Dow JA (2013). Data-mining the FlyAtlas online resource to identify core functional motifs across transporting epithelia.. BMC Genomics.

[R7] Duivenvoorden WC, Paschos A, Hopmans SN, Austin RC, Pinthus JH (2014). Endoplasmic reticulum protein ERp46 in renal cell carcinoma.. PLoS One.

[R8] Farkas R, Sutáková G (1998). Ultrastructural changes of Drosophila larval and prepupal salivary glands cultured in vitro with ecdysone.. In Vitro Cell Dev Biol Anim.

[R9] Farkas R, Suáková G (1999). Developmental regulation of granule size and numbers in larval salivary glands of drosophila by steroid hormone ecdysone.. Cell Biol Int.

[R10] Kuraishi T, Nakagawa Y, Nagaosa K, Hashimoto Y, Ishimoto T, Moki T, Fujita Y, Nakayama H, Dohmae N, Shiratsuchi A, Yamamoto N, Ueda K, Yamaguchi M, Awasaki T, Nakanishi Y (2009). Pretaporter, a Drosophila protein serving as a ligand for Draper in the phagocytosis of apoptotic cells.. EMBO J.

[R11] Loganathan R, Kim JH, Wells MB, Andrew DJ (2020). Secrets of secretion-How studies of the Drosophila salivary gland have informed our understanding of the cellular networks underlying secretory organ form and function.. Curr Top Dev Biol.

[R12] Ma CJ, Yang Y, Kim T, Chen CH, Polevoy G, Vissa M, Burgess J, Brill JA (2020). An early endosome-derived retrograde trafficking pathway promotes secretory granule maturation.. J Cell Biol.

[R13] Mann H. B., Whitney D. R. (1947). On a Test of Whether one of Two Random Variables is Stochastically Larger than the Other. The Annals of Mathematical Statistics.

[R14] Nakano R, Iwamura M, Obikawa A, Togane Y, Hara Y, Fukuhara T, Tomaru M, Takano-Shimizu T, Tsujimura H (2019). Cortex glia clear dead young neurons via Drpr/dCed-6/Shark and Crk/Mbc/dCed-12 signaling pathways in the developing Drosophila optic lobe.. Dev Biol.

[R15] Øvrebø JI, Edgar BA (2018). Polyploidy in tissue homeostasis and regeneration.. Development.

[R16] Prieto D, Aparicio G, Morande PE, Zolessi FR (2014). A fast, low cost, and highly efficient fluorescent DNA labeling method using methyl green.. Histochem Cell Biol.

[R17] Reactome Pathway Database. 2024. Lysosome Cargo [lysosomal lumen] (Stable Identifier: R-DME-435030). From https://reactome.org.

[R18] Rizki TM (1967). Ultrastructure of the secretory inclusions of the salivary gland cell in Drosophila.. J Cell Biol.

[R19] Robinson SW, Herzyk P, Dow JA, Leader DP (2012). FlyAtlas: database of gene expression in the tissues of Drosophila melanogaster.. Nucleic Acids Res.

[R20] Schindelin J, Arganda-Carreras I, Frise E, Kaynig V, Longair M, Pietzsch T, Preibisch S, Rueden C, Saalfeld S, Schmid B, Tinevez JY, White DJ, Hartenstein V, Eliceiri K, Tomancak P, Cardona A (2012). Fiji: an open-source platform for biological-image analysis.. Nat Methods.

[R21] Shapiro S. S., Wilk M. B., Chen H. J. (1968). A Comparative Study of Various Tests for Normality. Journal of the American Statistical Association.

[R22] Smith AV, Orr-Weaver TL (1991). The regulation of the cell cycle during Drosophila embryogenesis: the transition to polyteny.. Development.

[R23] Zhimulev I, Vatolina T, Levitsky V, Tsukanov A (2024). Developmental and Housekeeping Genes: Two Types of Genetic Organization in the Drosophila Genome.. Int J Mol Sci.

